# High-density transcranial direct current stimulation to improve upper limb motor function following stroke: study protocol for a double-blind randomized clinical trial targeting prefrontal and/or cerebellar cognitive contributions to voluntary motion

**DOI:** 10.1186/s13063-023-07680-8

**Published:** 2023-12-04

**Authors:** Xavier Corominas-Teruel, Martina Bracco, Montserrat Fibla, Rosa Maria San Segundo, Marc Villalobos-Llaó, Cecile Gallea, Benoit Beranger, Monica Toba, Antoni Valero-Cabré, Maria Teresa Colomina

**Affiliations:** 1 Sorbonne Université, Institut du Cerveau - Paris Brain Institute - ICM, Groupe de Dynamiques Cérébrales, Plasticité Et Rééducation, FRONTLAB Team, Inserm, CNRS, APHP, Hôpital de La Pitié Salpêtrière, Paris, France; 2 Department of Psychology and Research Center for Behaviour Assessment (CRAMC), Universitat Rovira I Virgili, Neurobehaviour and Health Research Group, NEUROLAB, Tarragona, Spain; 3 Sorbonne Université, Institut du Cerveau - Paris Brain Institute - ICM, Movement Investigation and Therapeutics Team, MOVIT Team, Inserm, CNRS, APHP, Hôpital de La Pitié Salpêtrière, Paris, France; 4 Rehabilitation and Physical Medicine Department, Hospital Universitari Joan XXIII, Tarragona, Spain; 5 Sorbonne Université, Institut du Cerveau - Paris Brain Institute - ICM, Centre de Neuro-Imagerie de Recherche, CENIR, Inserm, CNRS, APHP, Hôpital de La Pitié Salpêtrière, Paris, France; 6 Dept. Anatomy and Neurobiology, Lab of Cerebral Dynamics, Boston University School of Medicine, Boston, USA; 7 Cognitive Neuroscience and Information Tech. Research Program, Open University of Catalonia (UOC), Barcelona, Spain

**Keywords:** Stroke, Transcranial direct current stimulation, Plasticity, Neurorehabilitation, Randomized controlled trial

## Abstract

**Background:**

Focal brain lesions following a stroke of the middle cerebral artery induce large-scale network disarray with a potential to impact multiple cognitive and behavioral domains. Over the last 20 years, non-invasive brain neuromodulation via electrical (tCS) stimulation has shown promise to modulate motor deficits and contribute to recovery. However, weak, inconsistent, or at times heterogeneous outcomes using these techniques have also highlighted the need for novel strategies and the assessment of their efficacy in ad hoc controlled clinical trials.

**Methods:**

We here present a double-blind, sham-controlled, single-center, randomized pilot clinical trial involving participants having suffered a unilateral middle cerebral artery (MCA) stroke resulting in motor paralysis of the contralateral upper limb. Patients will undergo a 10-day regime (5 days a week for 2 consecutive weeks) of a newly designed high-definition transcranial direct current stimulation (HD-tDCS) protocol. Clinical evaluations (e.g., Fugl Meyer, NIHSS), computer-based cognitive assessments (visuo-motor adaptation and AX-CPT attention tasks), and electroencephalography (resting-state and task-evoked EEG) will be carried out at 3 time points: (I) Baseline, (II) Post-tDCS, and (III) Follow-up. The study consists of a four-arm trial comparing the impact on motor recovery of three active anodal tDCS conditions: ipsilesional DLPFC tDCS, contralesional cerebellar tDCS or combined DLPFC + contralesional cerebellar tDCS, and a sham tDCS intervention. The Fugl-Meyer Assessment for the upper extremity (FMA-UE) is selected as the primary outcome measure to quantify motor recovery. In every stimulation session, participants will receive 20 min of high-density tDCS stimulation (HD-tDCS) (up to 0.63 mA/${\mathrm{cm}}^{2}$) with ${\mathrm{\pi cm}}^{2}$ electrodes. Electrode scalp positioning relative to the cortical surface (anodes and cathodes) and intensities are based on a biophysical optimization model of current distribution ensuring a 0.25 V/m impact at each of the chosen targets.

**Discussion:**

Our trial will gauge the therapeutic potential of accumulative sessions of HD-tDCS to improve upper limb motor and cognitive dysfunctions presented by middle cerebral artery stroke patients. In parallel, we aim at characterizing changes in electroencephalographic (EEG) activity as biomarkers of clinical effects and at identifying potential interactions between tDCS impact and motor performance outcomes. Our work will enrich our mechanistic understanding on prefrontal and cerebellar contributions to motor function and its rehabilitation following brain damage.

**Trial registration:**

ClinicalTrials.gov NCT05329818. April 15, 2022.

**Supplementary Information:**

The online version contains supplementary material available at 10.1186/s13063-023-07680-8.

## Introduction

Middle cerebral artery (MCA) strokes are known to cause direct structural damage to key sensory-motor networks in charge of executing and controlling voluntary motion actions in frontal and anterior parietal cortical or in associated subcortical structures. Despite the influence of lesion location and extent [1–3], the magnitude of motor performance deficits following stroke [4–6] cannot be solely explained by ischemic damage on motor systems but also by diaschetic effects altering network interactions with local and distant structures contributing substantially to optimal motor activity.

Transcranial direct current stimulation (tDCS) is among the most popular non-invasive brain stimulation approaches currently used in clinical settings. It is characterized by its portability, low cost, ease of use, a safe profile of side effects, and high flexibility to target several locations simultaneously. Transcranial DCS is based on the application of a low-intensity continuous current inducing polarity-dependent sub-threshold shifts of neuronal resting membrane potential towards (anodal) or away (cathodal) from the firing threshold [7–10].

By virtue of such effects, whereas single sessions of tDCS have shown the ability to transiently modulate corticospinal excitability, periodical sessions of such simulation promote long-term potentiation/depression-like plasticity [11]. Transcranial DCS has been used as a therapeutic intervention to boost cognitive and motor recovery following stroke in diverse settings and evaluated with regards to its ability to improve voluntary upper limb function [12–16]. Despite its success in small samples of selected participants, the effects reported by accumulative tDCS interventions in stroke have often been shown to be inconsistent when applied to larger cohorts of patients [17]. Additionally, tDCS outcomes have been found to be highly influenced by variables such as post-stroke-to-treatment-onset time-lag, lesion site and volume, a large variety of stimulation parameters (electrode location, current intensity, density, regime periodicity, etc.), and interindividual head and brain anatomical differences affecting the distribution of tDCS-generated electric fields [18, 19].

Over the last decade, classical non-invasive brain stimulation (NIBS) approaches with transcranial magnetic stimulation (TMS) or with tDCS have privileged clinical strategies based on either the upregulation of directly affected primary motor systems or the downregulation of homologue motor networks of the spared contralesional hemisphere linked with the former via inhibitory trans-callosal inter-hemispheric projections.

Nevertheless, the neural signature of MCA lesions has revealed strong interactions with alterations in sustained attention (engaging prefrontal and fronto-parietal systems) and large-scale desynchronization phenomena, both key factors limiting motor function or precluding recovery after brain damage [20–22]. More specifically, the severity of motor deficits has been associated with the strengthening of impaired inter-hemispheric functional connectivity between the dorsal attention network (DAN) and sensory-motor networks (SMN) [23–26] and high- and low-frequency oscillatory abnormalities (decreases and increases respectively) in the injured hemisphere [27, 28]. Likewise, a connectome-based predictive model exploring fractional anisotropy (FA) in stroke has highlighted the role of the ipsilesional dorsolateral prefrontal cortex (DLPFC) and cerebellar areas subtending motor symptoms and suggested their potential to convey motor recovery when manipulated with NIBS interventions [29]. In such a context conventional therapeutic neurostimulation directly modulating the excitability of damaged areas in charge of mapping lost functions has shown inconsistent outcomes. Heneforth, recent innovative approaches [30, 31] have been focused on reversing abnormal large-scale network signatures by inducing synergistic effects from spared cortical regions contributing to impaired function [30, 31].

On such basis and superseding the clinical limitations of traditional approaches [32–34], we here aim at providing evidence that a multi-site tDCS stimulation approach targeting simultaneously the dorsolateral prefrontal cortex and the anterior lobe of the cerebellum will be able to drive significant clinical improvement in upper limb motor function compared to a placebo intervention, and also that such effects would be greater than the isolated stimulation of either site individually. Importantly, a battery of secondary measures relying on cognitive tasks assessing cerebellar and prefrontal contributions to motor function, sustained and selective attentional processes, EEG recordings and structural MRI neuroimaging will inform on some of the neural mechanisms involved in such recovery.

## Study objectives

The main and primary “key” objective pursued in this clinical trial is to (1) identify the clinical potential of the multitarget cortical stimulation by comparing the effect of three tDCS interventions (anterior cerebellar lobe stimulation, dorsolateral prefrontal stimulation and the combination thereof) driving clinical improvements of upper limb motor function in stroke patients, as assessed by the Fugl-Meyer Assessment (FMA). We specifically hypothesize (key hypothesis of the study) that combined stimulation of the former two cerebral sites will enhance clinical recovery compared to sham stimulation and also to the stimulation of either site (anterior cerebellar or dorsolateral prefrontal) in isolation.

The secondary objectives of our clinical trial are: (2) to evaluate the influence of such interventions on specific cognitive and motor processes sensitive to the contribution of the modulated targets, i.e., via an impact on sustained attention and cognitive control (dorsolateral prefrontal tDCS) or motor adaptation skills (anterior cerebellar tDCS); (3) to explore whether clinical improvements are induced by isolated interventions or if their combination can be associated to the normalization of neurophysiological outcome measures (increased local and/or inter-areal synchronization or changes of abnormal neural states, taken as proxies of enduring adaptive plasticity); finally, (4) to identify biomarkers (clinical and cognitive scores, neuroimaging features, and electrophysiological measures and tDCS current distribution modelled parameters) associated to the severity of motor impairments and their improvement following stimulation.

## Material and methods

### Study settings

This trial will be conducted in the *Hospital Universitari Joan XXIII*, Tarragona, Spain. All interventions and assessments will be carried out in the Rehabilitation and Physical medicine department of this hospital. MRI acquisitions will be performed in the Radiology and Nuclear medicine department in this same clinical institution. An IRB protocol in accordance with the Declaration of Helsinki covering all its procedures and interventions with the registration number: 077/2021 (version: V.1_06/05/) has been officially approved on June 27th 2021 by the local ethics committee of the *Institut d’investigació sanitària Pere Virgili* (IISPV, Tarragona, Spain).

### Study design

The present clinical protocol (hereafter entitled *E-Brain*) is designed as a double-blind, parallel, sham-controlled, randomized clinical trial.

### Study summary

**Fig. 1:**
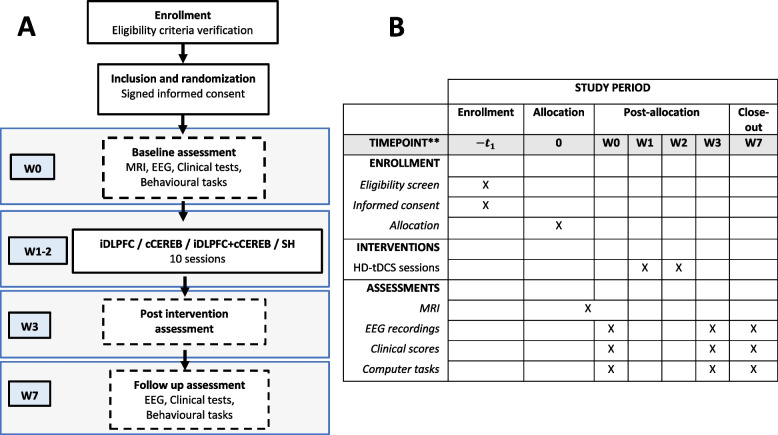
**A** Flow diagram of the study design. The diagram presents a weekly calendar where W0 denotes the baseline assessment and the interventional starting point for a representative subject. contra-CEREB: anodal contralesional anterior cerebellar lobe stimulation, ipsi-DLPFC + contra-CEREB: anodal ipsilesional DLPFC tDCS combined with anodal contralesional anterior cerebellar lobe simultaneous tDCS, ipsi-DLPFC: anodal ipsilesional dorsolateral prefrontal tDCS, SHAM: sham tDCS stimulation, W0: week 0 (baseline assessment), W1-2: weeks 1 and 2 (interventional weeks were the 10 days tDCS treatment is executed), W3: week 3 (post-intervention assessment), W7: week 7 (follow-up assessment). **B** Standard protocol items: recommendations for interventional trials (Supplementary file 1). Schematic summary and milestones for enrolment, tDCS treatment, and assessments across the study time-line

Participants will be randomly assigned to one out of the following 4 groups, GROUP 1: (ipsi-DLPFC) anodal tDCS stimulation of the ipsilesional dorsolateral prefrontal cortex; GROUP 2: (contra-CEREB): anodal stimulation of the contralesional cerebellar cortex (anterior lobe); GROUP 3: (ipsi-DLPFC + contra-CEREB) combined anodal stimulation of the ipsilesional DLPFC and the anodal contralesional cerebellar cortex; and GROUP 4: (SHAM tDCS) consisting in sham/placebo stimulation (see Fig. 1A).

For all groups, a daily tDCS session will be administrated for 10 consecutive days, with a regime of 5 sessions per week for 2 weeks (Monday to Friday). A conventional clinical magnetic resonance imaging (MRI, 3D-T1) recorded between the 1st and 3rd month after the stroke event will be retrieved from the participant’s medical history or recorded at the the Hospital Universitari Joan XXIII, Tarragona (Spain) within 2 weeks prior to the trial onset. Clinical scales or scores, behavioral computer-based tasks, and electroencephalography (EEG) evaluations will be carried out at 3-time points or milestones: (I) Baseline assessment: 72–96 h (2–3 days) before the onset of the tDCS regime; (II) Post-tDCS assessment: 72–96 h (2–3 days) after the end of the tDCS regime; and (III) Follow-up assessment: 30 days after the end of the tDCS regime. Across sessions, all evaluations will be carried out under identical conditions (see Fig. 1A, B). The current protocol E-Brain follows the SPIRIT recommendations (see Additional file 1).

### Participant recruitment

Participants will be enrolled through the Rehabilitation and Physical Medicine department of the *Hospital Universitari Joan XXIII* in Tarragona, Spain. Only those patients attending the rehabilitation service enrolled in an active rehabilitation program (hereafter referred to as a “live” rehabilitation program) will be assessed for eligibility. An experienced licensed medical doctor (RMS) working for the protocol will initially screen potential participants fulfilling eligibility criteria. Following verification of inclusion criteria, participants willing to participate will be presented with the details of the protocol and asked to sign a consent form to be officially included in the study.

### Inclusion criteria

 The following inclusion criteria have been established as to be fulfilled by potential participants: (1) to have received a diagnosis of supratentorial ischemic or hemorrhagic unilateral stroke supplied by the middle cerebral artery (i.e., encompassing frontal–temporal-parietal regions); (2) to be enrolled in a “live” rehabilitation program in the Rehabilitation and Physical Medicine department of our institution; (3) to be between 18 and 85 years old; (4) to have suffered a stroke within 4 and 12 months prior to enrollment; (5) to have signed the informed consent form.

### Non-inclusion criteria

Stroke participants presenting at least one of the following criteria will not be able to participate in our study: (1) unstable medical condition (e.g., affected by infections, with assisted ventilation, having suffered or actively suffering epilepsy or recurrent seizures, untreated psychiatric disorders, or being under an active treatment with sedative drugs); (2) participants presenting contraindications to tDCS according to the most current international NIBS safety guidelines [10]; (3) participants presenting cognitive impairments-such as severe aphasia or neuropsychiatric deficits-limiting their comprehension and their ability to follow instructions. The verification of non-inclusion criteria will be documented by means of an in-house screening questionnaire.

### Exit criteria

Patients will be dropped out from the study if: (1) they manifest, at any time and without the need to provide any explanation, their willingness to stop their participation in the clinical trial; (2) they are not compliant with the procedures of the study; (2) they experience severe discomfort or annoyance during their participation (i.e., insomnia, headache, etc.); or (3) in case of unexpected events that incapacitate patients to continue in the study. Data collected until a participant is officially considered a 'drop-out' will be included in analyses, hence not withdrawn from the study.

### Sociodemographic data

During the baseline assessment, patients will be asked to complete to the best of their knowledge and ability a questionnaire including the following information: (1) age and sex; (2) stroke features (hemisphere affected, localization, stroke type, time since stroke event, pre-morbid conditions); (3) socio-educational information (marital status, academic level, occupation, leisure hobbies, technology usage, sport practice, smoking, alcohol or drug usage and ongoing medication); (4) past and ongoing stroke rehabilitation program (post-stroke-onset time, types of ongoing and completed programs, frequency, intensity, and periodicity). Stroke lesion features, details of the clinical history, and any missing medical information required to complete the above-mentioned questionnaire will be verified and/or completed by a legally authorized member of the medical team collaborating with the protocol (co-author RMS).

Additionally, the Edinburgh manual dexterity scale and the Beck’s inventory will be administered to characterize the patient’s laterality and assess the participant’s mood, respectively.

### Sample size

The current study is designed as a pilot clinical trial aiming to assess the effects of an isolated monofocal (single cortical site stimulation, i.e., either the dorsolateral prefrontal cortex or the anterior cerebellar lobe) or combined multifocal (simultaneous stimulation of the two former sites) multiday tDCS intervention using a high-density array of electrodes. Given the exploratory character of our study, no power analysis to estimate sample size was required by the local IRB committee. Instead, following standardized guidelines for experimental clinical trials, we considered the number of participants included in past similar exploratory tDCS studies warranting sufficient statistical power [35] and ensuring recruitment feasibility, and we concluded the need to include at least *n* = 15 patients in each of our 4 tDCS treatment groups.

### Randomization and blinding

The study will include *n* = 60 patients with chronic MCA stroke, randomly assigned to one of the 4 experimental groups indicated above (ipsi-DLPFC, contra-CEREB, ipsi-DLPFC + contra-CEREB, or SHAM tDCS). A patient randomization algorithm (*MinimPy* software running in *Python environment*, https://sourceforge.net/projects/minimpy/) counterbalancing groups by sex (Woman/Male), age (− 65 years/ + 65 years) and stroke type (ischemic/hemorrhagic) will ensure equivalence for these three variables across the 4 experimental groups. The biased coin method is an algorithm implementing a biased coin minimization algorithm (base probability: 1, allocation ratio 1:1:1:1) [36] for sequential dynamic allocation, in which each new allocation is influenced by the current state of balance across delivered treatments [37].

An independent co-investigator not involved in the recruitment/enrollment of patients, tDCS application or in clinical evaluation will be in charge of patient randomization and condition/group allocation. The groups of patients associated with the four randomized tDCS conditions will be assigned a letter-coded name (Groups A, B, C, or D) providing no clue with regard to the ultimately delivered tDCS strategy. This same code will be used at all times for patient allocation and only an independent co-investigator in charge of randomization/allocation activities will be able to associate each of the codes (A, B, C, and D) to a specific tDCS condition (aka treatment group). Once a patient will be randomly assigned to a condition/treatment group, its code (A, B, C, or D) will be communicated to a team member in charge of stimulation which will simply deliver it blindly in a non-identifiable manner. To search for the potential influence of placebo or nocebo effects, investigators will debrief at the end of the follow-up, with patients and ask them to guess which stimulation group they believed they had been allocated to (allocation perception).

Double-blind (both the participant and the investigators in charge of stimulation or evaluation tasks will be unaware of the stimulation condition) will be ensured by a *blinding option* available on tDCS equipment and associated control software (*Starstim-8*® and *Neuroelectrics Instrument Controller*®, Neuroelectrics, Barcelona, Spain)*.* An investigator (co-author MF, referred hereafter as the 'administrator') will program and blind the different HD-tDCS protocols in our *Neuroelectrics Instrument Controller* system, whereas a second researcher (co-authors XC-T, MV-L MTC, or AV-C, referred to as the 'operator') will perform the intervention sessions without knowledge of the tDCS protocol being delivered. Likewise, the operator will be in charge of EEG and (both the participant and the investigators in charge of stimulation or evaluation will be unaware of the stimulation condition) assessment performance. Finally, data analysis will be performed by a fourth co-investigator who will remain blind to the specific tDCS strategies implemented in each treatment condition (A, B, C, or D) until all analysis work is completed.

### Interventions

#### tDCS electrode montage

Prior to the design of the present clinical Trial, we optimized a HD-tDCS electrode montage solution to be able to target simultaneously the DLPFC and the anterior lobe of the cerebellum using an 8-channel tDCS (Starstim, NE) equipment. The optimized computational model was generated in MATLAB (R2019a, Mathworks, USA) and SimNIBS 3.2.3 [38], an open-source package for the simulation of non-invasive brain stimulation electrical field based on participant’s MRI volumes using a standard head/brain volume MNI152 (version 2009a) as template (available through the open dataset of SimNIBS). The “lead field matrix” computation defining the scalp localization of tDCS electrodes was based on the 10/20 EEG system and ${\mathrm{\pi cm}}^{2}$ predefined tDCS electrode size. The MNI152 standard head model was reconstructed with the *headreco* routine relying on SPM12 and CAT12 for segmentation. Isotropic conductivity values were set as follows (in S/m) based on previous studies [39, 40]: WM 0.126, GM 0.275, CSF 1.654, bone 0.010, scalp 0.465, eye balls 0.500, compact bone 0.008, spongy bone 0.025, blood 0.600, muscle 0.160. The selected electrode solution (in terms of electric field focality, intensity, and electrode compatibility with an 8-channel tDCS *NE Starstim* device) was obtained by optimizing the electrode positions of each cerebral target separately (ipsi-DLPFC and contra-CEREB) and merging these solutions in a simulation of combined stimulation scenario (ipsi-DLPFC + contra-CEREB).

For an optimal simulation of the left (ipsilateral) DLPFC (MNI *x* =  − 39 *y* = 34 *z* = 37 to influence the Dorsal Attentional Network, DAN), we obtained the best solution limiting the total number of electrodes to 3. Additionally, given technical limitations of our ISO and CE certified tDCS device and to warrant patient safety, a total maximal current of 4 mA and a maximal individual electrode current of 2 mA were used. Ipsilesional prefrontal target coordinates (ipsi-DLPFC) were defined on the basis of previous studies ensuring a high E-field impact in Brodmann area 46 (BA46) [41]. For the computation of the optimal right contralateral cerebellum stimulation site (MNI *x* =  24 *y* =  − 66 *z* =  − 40) and to optimally impact its anterior lobe (contra-CEREB), the best solution was found with a total number of 5 scalp electrodes, a maximum current of 4 mA, and maximum individual electrode current of 2 mA[42]. We also modified the E-field *direction* controlling field strength at the target instead of a tangential orientation. Importantly, to favor spatial selectivity and avoid electrode dispersion, the contralesional temporal cortex (MNI *x* =  53 *y* =  − 6 *z* =  − 41, hence contralateral to the stimulated DLPFC) and the ipsilesional anterior cerebellar lobe (MNI *X* = -9 *Y* =  − 88 *Z* =  − 44, contralateral to the stimulated CEREB) were defined as “avoidance” regions which tDCS montages excluded from being impacted.

**Fig. 2:**
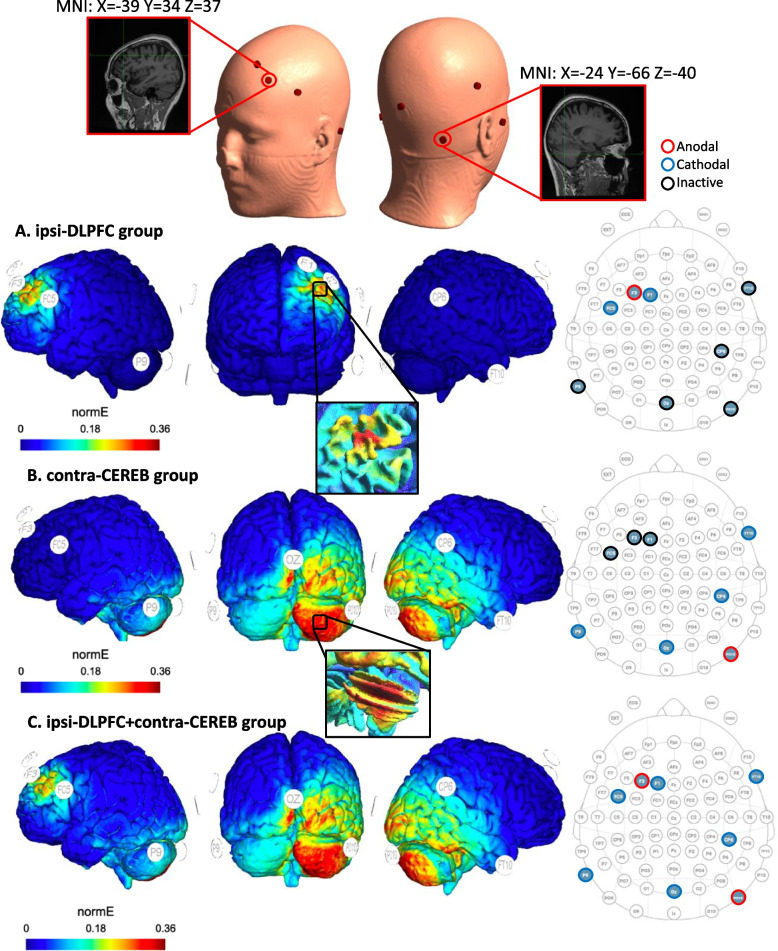
Computational biophysical models of an in-house ipsilateral dorsolateral prefrontal (ipsi-DLPFC), contralateral anterior cerebellar lobe (contra-CEREB), and combined prefronto-cerebellar (ipsi-DLPFC + contra-CEREB) tDCS montages were developed in SimNIBS 3.2.3 using a 3D reconstruction of the MNI152 model brain. Electrode positions surrounded by red circles represent active anodes; those surrounded by blue circles, correspond to active cathodes; finally, electrodes surrounded by black circles represent inactive electrodes (placed in the head cap during the stimulation only to ensure the operator’s blinding). **A** Optimized montage solution for the ipsilesional prefrontal target (DLPFC) assuming a left hemisphere stroke. Ag/AgCl $\uppi {\mathrm{cm}}^{2}$ electrode position and current intensities are defined by an optimization procedure resulting in the following scalp montage and currents to be delivered (10/20 EEG system): F3 (1.736 mA), F1 (− 1.222 mA), and FC5 (− 0.536 mA). **B** Illustration of the optimized solution for the contralesional anterior cerebellar lobe (CEREB) target assuming a left hemisphere stroke. Ag/AgCl $\uppi {\mathrm{cm}}^{2}$ electrode position on the scalp and intensities were automatically defined by an optimization procedure resulting in the following montage (10/20 EEG system): PO10 (1.960 mA), FT10 (− 0.550 mA), CP6 (− 0.180 mA), Oz (− 0.630 mA), and P9 (− 0.631 mA). **C** Final simulation merging DLPFC and cerebellar electrode montages assuming in this specific case a left hemisphere stroke, pertaining to the prefronto-cerebellar stimulated group. All images are presented in terms of total electric field strength (|E|). The visual color impact scale was normalized in all images from a minimum of 0 V/m (blue areas) up to a maximum of 0.36 V/m (red areas). Contra-CEREB: anodal contralesional cerebellum, ipsi-DLPFC: anodal ipsilesional dorsolateral prefrontal cortex, ipsi-DLPFC + contra-CEREB: anodal ipsilesional dorsolateral prefrontal cortex with anodal contralesional cerebellum, normE: electric field strength (|E|)

Current evidence from in vitro and in vivo studies has demonstrated that tDCS field strength close to 0.25 V/m are sufficient to alter neuronal excitability via modulation of its resting-state potential [9, 43, 44]. Due to the dual-site experimental approach tested in our study (simultaneous stimulation of two sites, cerebellar and prefrontal for the combined tDCS condition) and considering a maximum of 4 mA injected current with high-density electrode montages (current density up to 0.63 mA/${\mathrm{cm}}^{2}$), the optimization algorithm generated assuming a left hemisphere stroke (right ipsi-DLPFC and left contra-CEREB stimulation), suggested 0.25 V/m simultaneously to both targets. This intensity was also retained to avoid possible side effects and warrant the safety/tolerability of our electrode montage. The “flipped version” of the former montage, hence assuming a right hemisphere stroke (right ipsi-DLPFC and the left contra-CEREB stimulation) was also simulated and delivered the same optimized electrode solution (see Fig. 2 for details).

#### Stimulation procedures

HD-tDCS will be delivered to all experimental groups with a Starstim-8® device, a wireless hybrid EEG/tES 8-channel system (Neuroelectrics, Barcelona, Spain). The Neuroelectrics Instrument Controller® software (NIC) associated with this hardware will be used to pre-program all HD-tDCS and EEG protocols and carry out interventions. Given the four-parallel arms of our study design (ipsi-DLPFC, contra-CEREB, ipsi-DLPFC + contra-CEREB, and SHAM) and considering participants with left or right hemisphere stroke, a total of 8 HD-tDCS protocols will be pre-programmed.

During stimulation sessions, participants will wear a neoprene cap adapted to the circumference of their head ensuring correct placement for NG pistim® electrodes (πcm^2^ surface, Ag/AgCl) embedded in SignaGel® (Parker laboratories, USA) to keep impedances below 10 KΩ. Every participant will receive 10sessions of tDCS, 20 min each and 5 daily sessions per week (Monday to Friday) during 2 consecutive weeks (W1 and W2 of participants’ schedule).

**Fig. 3:**
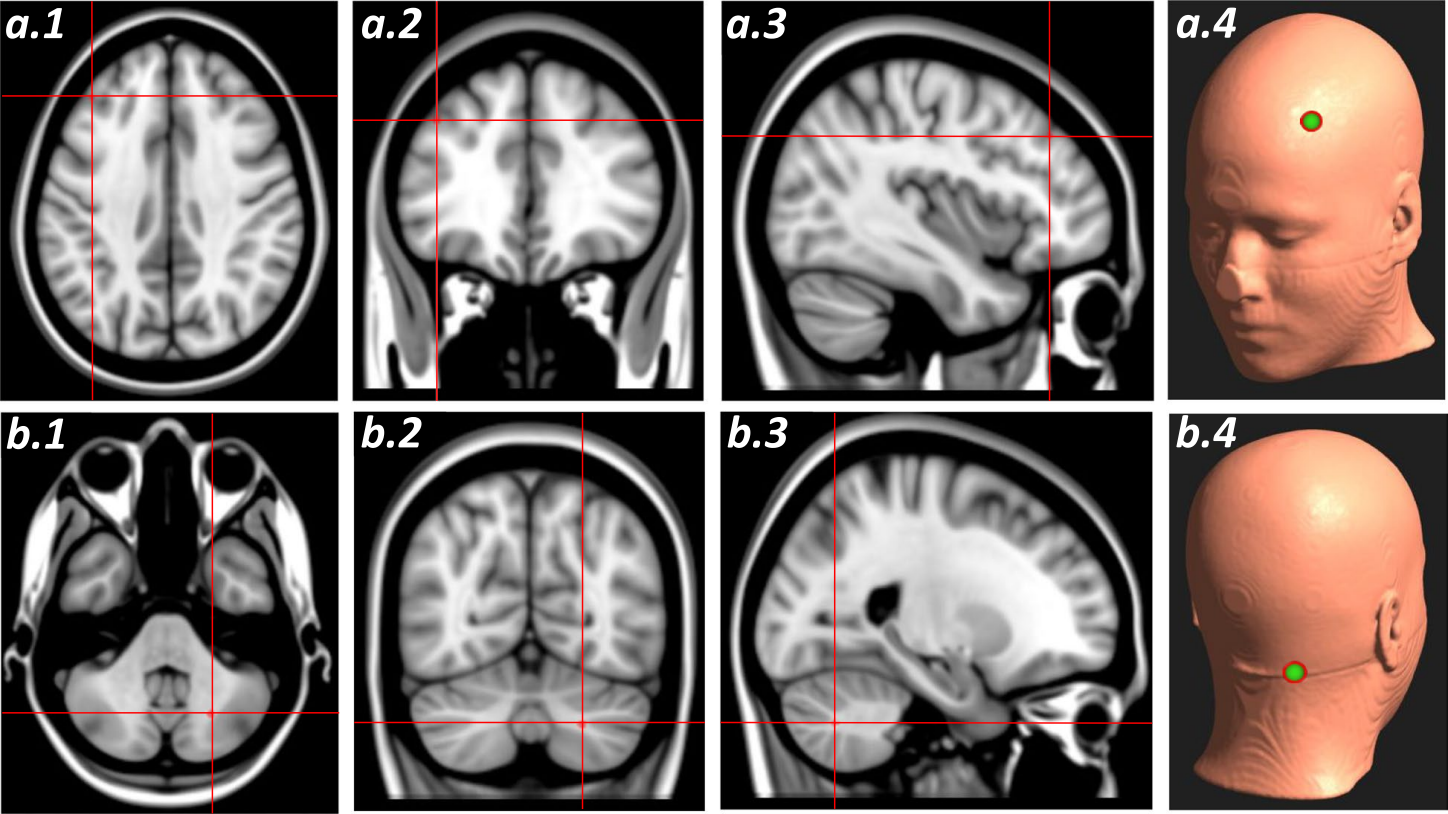
Transcranial tDCS targeted regions (used for electrode optimization and subsequent stimulation) assuming in the image a left hemisphere stroke. Established MNI (Montreal Neurological Institute) coordinates have been selected ensuring a constant electric field impact (0.25 mA) in Brodmann area 46 for the dorsolateral prefrontal cortex (DLPFC) and lobules  I–IV of theanterior cerebellar lobe (CEREB). **A** DLPFC target area (MNI coordinates *X* =  − 39, *Y* = 34, *Z* = 37) in an axial (*a.1*), coronal (*a.2*) sagittal (*a.3*) MRI sections and their scalp projection (*a.4*) views. **B** Cerebellar target area (MNI coordinates: *X* =  24, *Y* =  − 66, *Z* = 40) in axial (*b.1*), coronal (*b.2*), sagittal (*b.3*) MRI sections and their scalp projection (*b.4*) views. Target areas are represented in the MNI152 and labelled on a standard MRI model by means of an MRI-based frameless stereotaxic neuronavigation system (Brainsight)

The tDCS device integrates a maximum of 8 electrodes to deliver stimulation. However, note that depending on the experimental tDCS group, different electrodes will be actively involved in stimulation at each condition (see tDCS montage protocol section). To conceal the stimulation condition and preserve the blinding of the operator and the participant, all 8 available electrodes will always be positioned on the participant’s head cap and will have their impedance tested. Crucial values such as mean voltage, current intensity, and impedance of the different electrodes employed will be recorded automatically during the sessions. Targeted regions are represented in Figs. 2 and 3.

#### Active tDCS conditions

Once the head cap is positioned and all the necessary electrodes are attached, the current intensity will be linearly increased (ramp up) for 30 secs to reach the peak current intensities delivered in each specific condition: (1) 1.736 mA intensity (0.55 mA/cm^2^ density) in the ipsi-DLPFC group (receiving ipsilesional prefrontal stimulation); (2) 1.999 mA intensity (0.63 mA/cm^2^ density) in the contra-CEREB group (receiving contralesional anterior cerebellar lobe stimulation); and (3) 3.735 mA intensity (0.55 mA/cm^2^ density in the ipsi-DLPFC area and 0.63 mA/cm^2^ in contra-CEREB) in the ipsi-DLPFC + contra-CEREB condition (receiving simultaneously dorsolateral prefrontal and cerebellar stimulation). Once the predefined intensity is reached, the current will be kept active for 20 min, a treatment duration that has been proven effective in prior studies with neurological patients [14, 15]. Finally, tDCS current will be ramped down for 30 secs at the end of the stimulation session.

During the delivery of tDCS, patients will be seated in a comfortable chair. To keep patients awake and restrict interindividual and intraindividual variability caused by a diversity of neural states present across subjects and sessions, participants will be asked to perform a simple computer-based game on a monitor placed 57 cm in front of them (hence at arm’s reach) requiring keyboard presses (space bar) with their non-impaired hand, every time a moving dot contacts the borders of a centered rectangular placeholder (8 × 13 degrees of angle/cm).

#### Sham tDCS condition

The sham tDCS intervention will follow the same procedure as the active conditions, even though placebo current stimulation will be applied. At the beginning of the session, during the first 30 secs, electrical current will be ramped up emulating the ipsi-DLPFC + contra-CEREB stimulation condition. Immediately thereafter, a 5-secs ramp-down will cease the delivery  of electrical current. During the following 20 min of the session no electrical current will be administered. At the end of the session, the same procedure will be repeated, delivering a 5-secs ramp up followed by a 30-secs ramp down to null intensity. This protocol commonly known as “FISSFO” (Fade In of Stimulation, brief real Stimulation, Fade Out) has been extensively used in clinical trials to mimic during sham conditions the tingling and itching skin sensations perceived during the ramp up and ramp down of tDCS intensity [45]. In the current clinical trial, patients will undergo tDCS treatment during the late subacute to chronic phase following a stroke, hence optimal for non-invasive neuromodulation, but during which, instances of spontaneous recovery might still operate. Moreover, for ethical reasons, while participating in our study, patients will continue their conventional physical rehabilitation program (see next section for details), which *per se* could also contribute recovery. In such circumstances, and also to counter improvement effects caused by familiarization and training in evaluation tasks performed several times during the study follow-up, a sham stimulation group is key to identify genuine recovery fostered by transcranial stimulation.

#### Associated clinical rehabilitation

In order not to undermine optimal recovery potential and since we are testing an innovative tDCS protocol for which clinical efficacy is not warranted, all participants will be enrolled in parallel, in a similar “live” on-site rehabilitation program. Stimulation sessions will be conducted in the morning (8–12 am) and “live” rehabilitation sessions delivered independently in the same institution will follow. Rehabilitation activities during participation in the trial aim to maximize the chances of optimal recovery given the uncertain therapeutic value of our intervention and were specifically requested by our local Institutional Review Board for ethical reasons.

The on-site “live” program is based on an intense multidisciplinary rehabilitation plan encompassing specific goal-directed qualitative interventions, combining physical therapy, occupational therapy, neuropsychological, and speech therapy interventions, with a duration and periodicity of 1 to 2.5 hours a day, 2–3 days a week. Hence, all participants will conduct equivalent rehabilitation activities based on identical goal-directed qualitative occupational principles. On stimulation days, when hospital rehabilitation cannot be performed live on-site, participants will be instructed to carry out 1–2 hours of rehabilitation at home based on the same activities usually performed in the hospital. Clinical physical therapy is mainly focused on gross motor functions and postural control training (i.e., reaching, straightening, and support abilities), somatosensory integration, and gait rehabilitation. Occupational therapy exercise upper limb fine motor function, spasticity reduction, manual skill training, and multisensory stimulation. Finally, neuropsychological rehabilitation is mainly centered on training executive function and the management of emotions (i.e., impulsivity, liability, childish behavior, apathy, orientation and depression).

#### Safety monitoring

No major side effects of stimulation or the follow-up of stroke patients are to be expected given the well-known safety profile of tDCS interventions in neuropsychiatric patients. Nonetheless, in order to monitor safety and to evaluate tolerance and comfort to tDCS, participants will complete before and immediately following each stimulation session, a standardized adverse effect questionnaire [10] documenting the incidence and intensity of the most common side effects of tDCS stimulation (notably itching, pain, burning, fatigue and headache). Even if extremely unlikely, in case of a major adverse event-related or unrelated to stimulation such as for example a second stroke or an epileptic seizure participants will cease participation in the study and will be immediately provided with medical assistance and follow-up care in our institution.

### Outcome measures

In agreement with our objectives, the primary outcome measure (addressing the main and key objective) of our trial will evaluate changes in the Fulg-Meyer Assessment (FMA) for the impaired upper limb. A set of secondary outcome measures recorded prior to and following tDCS treatment (addressing study objectives 2, 3, and 4) will assess respectively: (2.1) Patient’s performance in a series of computer-based behavioral tasks evaluating visuo-motor adaptation (anterior cerebellar lobe contributions) and also sustained attention and cognitive control (prefrontal contributions); (2.2) Changes in resting-state and task-evoked EEG recordings and (2.3) set of clinical scales evaluating global stroke severity, cognitive impairment and their recovery, and correlations with predicted electric field distribution model features and stroke lesion hallmarks revealed by structural MRI neuroimaging. Find below a detailed explanation of the different outcome measures employed in our study.

#### Fugl-Meyer Assessment (FMA)

Addressing the primary or key goal of our study, the Fugl-Meyer Assessment (FMA) will be used to evaluate upper limb motor improvements. This is a widely employed tool used to assess motor impairment in post-stroke participants and considered one of the most comprehensive and reliable quantitative measures for motor hemiplegic dysfunction [46]. Among the sub-sections of this assessment we will focus on evaluating the upper limb/extremity motor domain (FMA-UE). Even so, the lower limb/extremity section of the assessment (FMA-LE) will be also administrated to evaluate the status and changes in lower limb motor function. The FMA-UE and FMA-LE include a series of items measuring movement, coordination and reflexes, each one scored on a 3-point ordinal scale (0 = cannot perform, 1 = performs partially, 2 = performs fully), with a total score of 0 points equaling absolute hemiplegia, and 100 points signalling sound motor function; of these 100 points, 66 are attributed to the upper extremity (FMA-UE) and 34 to the lower extremity (FMA-LE). For intra-subject pre-post-intervention assessments, an improvement greater than 6 points is usually defined as clinically significant.

#### Visuo-motor adaptation task

**Fig. 4:**
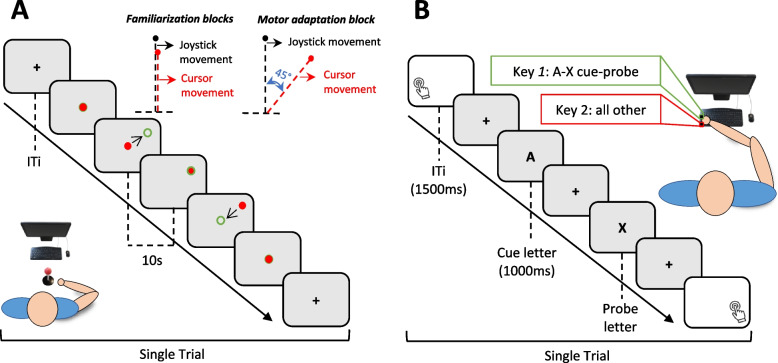
Computer-based selected tasks to assess cerebellar (motor learning and adaptation) and dorsolateral prefrontal (sustained attention and cognitive control) contributions to motor functions and their changes following stimulation. **A** Visuo-motor adaptation experimental paradigm design and setup. Following a stimulus (“ + ”), a red cursor representing the joystick position is displayed in the center of a computer screen. Participants are required to direct the cursor moving the joystick towards a randomly allocated target (within a time window of 10 secs) displayed as a green circle among other targets, equally spaced around a virtual circle. In the motor adaptation block, a force field with a 45° constant perturbation is applied deviating the trajectory of the cursor respect the actual joystick trajectory, hence forcing the participant to adapt and learn how to compensate such a deviation. **B** AX-CPT experimental design. Participants are instructed to attend to serial presentation of letters and to make a target response each time the correct 'cue-probe' (A + X) combination is presented (by pressing the key “1” on a keyboard), or execute an alternative response (by pressig the key “z”) for all other incorrect 'cue-probe' combinations (e.g., A + S, M + X). During the experimental recording, a total of 150 trials encompassing the presentation of a cue stimulus (1000 ms), a fixation stimulus “ + ” (1000 ms), and a probe stimulus (1000 ms) followed by an inter-trial interval (1500 ms) are completed. ITI: Inter-trial Interval

A visuo-motor adaptation task known to assess contributions from the anterior cerebellar lobe to voluntary motor function [47–50] programmed in a MATLAB environment (R2019a, Mathworks, USA) and Psycho-Toolbox will be implemented. During the task, participants will seat in a comfortable chair in an isolated room with no distractions at a distance of 57 cm (arm’s reach) in front of a 15-inch computer monitor, holding a hand-joystick with their impaired paretic upper limb. Hand splints to ensure correct attachment to the joystick will be employed if necessary for all subsequent measures to keep conditions constant. During the task, the joystick position will be displayed as a red dot of 1-cm diameter. Participants will be asked to complete a series of consecutive trials to direct the cursor (a red dot) from the center of the screen (starting point) towards the interior of a randomly allocated peripheral target displayed as a green circle. Each trial will start with the green circle and the red dot in the center of the screen. Subsequently, additional green circles (targets) will appear randomly in 4 possible locations, equally spaced around a virtual circle respecting a homogeneous 5 cm distance from the starting point. Participants will have 10 secs to move the red dot and place it inside the circle and maintain such position for 0.5 s. Once completed, the green circle will jump back to the starting point, and  participants had to replace the dot in the starting position to complete the trial. During the inter-trial interval (1.5 secs), a full gray screen with a center stimulus (“ + ”) will be displayed to keep participants alert and ready for a subsequent trial. If the trial is not successfully completed, hence the participant does not succeed in placing the dot inside the circle during the allotted time window, the circle will automatically jump to the starting point. Visual feedback of the dot and the circles will be displayed in real time (see Fig. 4A). The task will be divided into 2 consecutive blocks. The first block (familiarization) will consist of 32 trials, and will be followed by a second block (motor adaptation)including 64 trials. During the familiarization block, trials will be conducted as previously described. During the motor adaptation block, a “force-field-like” perturbation will be implemented by introducing a constant 45° angular rotation between the trajectory of the red dot trajectory and the real movement of the joystick, deviating the trajectory of the former and forcing participants to compensate for such shifts in order to reach the target in the alloted time, hence engaging visuo-motor adaptation (learning) skills.

#### AX continuous performance task

The AX continuous performance task (AX-CPT) is a computer-based paradigm running in E-prime software (E-Prime®, Psychology Software Tools, Sharpsburg, PA, USA) which has been extensively used to explore DLPFC contributions to sustained attention and cognitive control subtended by prefrontal systems. Moreover, performance in the task has been correlated with the severity of the motor impairment [51–53]. In this paradigm, participants will be comfortably seated at a distance of 57 cm from a 15-inch computer screen in an isolated room with no distractions, and required to attend to a serial presentation of letters and provide a response (press the “Q” key on the keyboard) every time the cue-probe letter combination “A” + “X” is presented. Likewise, participants are also instructed to execute an alternative response (press the “Z” key) when any cue-probe letter combination other than “A” + “X” is displayed on the screen (e.g., “A” + “S”). Participants are required to respond as quickly and accurately as possible after each cue-probe combination during a response interval using their non-impaired hand (please see Fig. 4B). Each trial consists of a cue letter stimulus (1000 ms duration), the “ + ” fixation stimulus (1000 ms duration), and a probe letter stimulus (1000 ms duration) followed by a 1500-ms response interval displaying a white screen. Letters on the screen will always be displayed in Times font (40 size), in black capital letters on a white background. The task will be split into two parts: first, (1) a series of practice trials acclimating the participant to the paradigm while receiving the researcher’s constant feedback to ensure participants correctly understand the task; second, (2) the experimental trials will be launched after a short rest (duration determine ad libitum by subject preferences). Twelve trials will be presented during a practice block followed by an experimental block including a total of 150 trials. Responses in both the practice and the experimental blocks will be recorded, but only the latter will be used for statistical analyses.

#### EEG acquisition

EEG data will be recorded with the same HD-tDCS equipment used for stimulation, a Starstim® 8 channel device controlled by the Neuroelectrics Instrument Controller® software (Neuroelectrics, Barcelona, Spain) able to sample scalp EEG signals at a frequency of 500 Hz using scalp NG pistim® electrodes (${\mathrm{\pi cm}}^{2}$ Ag/AgCl) and SignaGel® (Parker laboratories, USA). Skin/electrode impedance values will be automatically monitored and kept at all times below 5 KΩ. The 8 recording electrodes will be distributed across left and right hemi scalp positions (F3, F4, C3, C4, P3, P4, PO10, and PO9) according to the 10/20 EEG system, to capture among other sources, derived EEG activity associated to prefronto-central, and fronto-parietal motor/premotor networks. All EEG sessions will be conducted under the same conditions with a combined ground reference placed in the right earlobe. Ten minutes of resting-state EEG data (eyes open fixating on a target located at arm’s reach, ~ 57 cm) will be acquired during the baseline assessment, the post-stimulation regime assessment, and during the follow-up visit. EEG will be also continuously recorded during our visuo-motor adaptation task assessing cerebellar contributions to motor learning and during the AX-CPT paradigm assessing dorsolateral prefrontal contributions to sustained attention and cognitive control. Finally, 5 min of continuous resting-state EEG data (eyes open) will also be recorded prior to and following each of the 10 sessions of tDCS stimulation.

#### National institutes of health stroke scale

The National Institutes of Health Stroke Scale (NIHSS) is a quantitative scale of stroke-related neurologic deficits widely used to characterize baseline impairment in clinical trials [54]. It explores consciousness level, visual field surface, language function, the presence of hemineglect, hemiplegia, movement disorders, and impairments of sensory function. It consists of 15 items, each one scored from 0 to 4, to reach a total of 42 potential points, in which higher scores signal more severe impairment (0 = no stroke symptoms, 1–4 = minor impairment, 5–15 = moderate impairment, 16–20 = moderate to severe impairment, 21–42 = severe impairment). We will use the NIHSS to characterize the stroke severity of our patients and also use it to verify the comparability of each of the 4 experimental tDCS groups defined in our trial.

#### The Montreal cognitive assessment

The Montreal Cognitive Assessment (MoCA) is a quantitative screening tool used to explore stroke-related cognitive deficits [55]. It is made of a series of questions and tasks specifically designed to assess visuo-spatial and executive function, working memory, short-term memory, sustained attention, abstraction, language, and orientation in time and space. It contains a total of 10 items, for a total of 30 points, and lower scores indicate  higher levels of impairment (26–30 = no stroke cognitive impairment, 18–25 = mild cognitive impairment, 10–17 = moderate cognitive impairment, 0–10 = severe cognitive impairment). We will use the MoCA to evaluate participants’ cognitive impairment/improvement and also to asses group comparability.

#### Screening of hemispatial neglect

In order to monitor the presence of hemispatial neglect, participants will conduct a letter cancellation test, a Bells cancellation test, and a line bisection test. The letter cancellation test quantifies the presence of visuo-spatial  neglect and visual search/scanning deficits [56]. In the task, a total of *n* letters is distributed into 6 lines presented in paper format. Among them, the letter “H” is repeated 104 times. Participants will be asked to visually screen the paper sheet and find and outline as many letters “H” as they can find. The Bells cancellation test quantifies visual neglect deficits in the extra personal space [57]. A total of 35 bells are embedded within 280 distractors presented in paper format. Participants will be instructed to circle or cancel off all drawings corresponding to bells. All stimuli are of the same size and displayed in black color over a white background. Though they might appear randomly distributed, bells are presented in 7 columns; 3 columns on the left and the right hemifields and one in the middle, with 5 bells and 40 distractors on each one. The sheet of paper with the test display will be placed right in the middle of the visual field and the participant is free to explore the document with his/her gaze. Finally, the line bisection test identifies and quantifies visuo-spatial orienting impairments and spatial neglect deficits [58]. In this task, several examples of two types of black lines, 5 and 10 cm long, are presented on a white paper sheet placed in the middle of the participant’s visual field. Participants, who are free to explore stimuli with their  gaze, are instructed to identify the center of each horizontal line and bisect it with a pencil.

#### MRI imaging acquisition

MRI structural scans will be obtained from all stroke patients. These will be either drawn in anonymized coded form from recent recordings stored as clinical records by an authorized neurologist working for the protocol, or if not available, acquired  de novo within the 2 weeks before tDCS treatment onset. A 3-Tesla MRI scanner (Siemens Healthcare, Germany) located at the neuroradiology department at Hospital Universitari Joan XXIII in Tarragona will be used for image acquisition. In that case, a T1-weighted 3D anatomical sequence will be carried out  with the following parameters: repetition time = 2500 ms, echo-time = 2.12 ms, number of slices = 156, slice thickness = 0.94 mm, matrix size = 232 × 288, in-plane resolution = 0.83 mm × 0.83 mm, and flip angle = 9 °.

#### Data collection and management

All data generated by the study will be hosted in a secured internal server, property of the *Universitat Rovira i Virgili* (URV) in Tarragona. A shared encrypted database allowing access to anonymized datasets only to authorized co-investigators with a valid personal ID has been set up.Our clinical trial will generate three large classes of records: (1) demographic information, (2) clinical outcome datasets, and (3) neuroimaging and electrophysiological datasets including MRI scans and EEG recordings. Two computers, property of the URV, secured with personal encrypted passwords, will be used for databasing. Given the volume and complexity of the pre-processing involved in the analysis of MRI and EEG datasets, these datasets will be stored in specific computers handled by expert investigators associated to the project. To ensure data quality, all co-investigators and associated personnel taking part in the E-Brain trial will be trained by the principal investigators (co-authors XC-T, MT-C, and AV-C) to perform any of its procedures (screening, recruitment, informed consent, randomization and allocation, blinding, cognitive and clinical evaluations, EEG/MRI recordings, and tDCS application) by following strictly the conditions, criteria, materials, and methods established in the written protocol approved by our local IRB. In the same vein, all data will be collected by the same institution, and co-investigators will be specifically trained to strictly respect and implement established protocols for data collection, anonymization, storage, analytical strategies and “cloud” data management. To maintain at all times data anonymity and confidentiality, a reference number representing the participant until the study is terminated identifying individual datasets in our databases will be assigned to each participant at inclusion. Only co-authors XC-T, MT-C, and AV-C will be provided access to the full dataset generated by the study, with the exception of group allocation information which, as stated previously, will only be unblinded to these investigators, once data collection and analyses are completed.

## Data analyses

### Analysis of clinical and cognitive performance outcome measures

Outcomes extracted from computer-based tasks and clinical scores (see the previous section) will be analyzed to explore the differential ability of active tDCS conditions compared to sham stimulation to induce upper limb motor recovery following a stroke. For the clinical tests (FMA, NIHSS, MoCA, and hemispatial neglect tests), mean group scores post vs. pre-tDCS baseline intervention,and during the follow-up assessment, will be compared across experimental groups. Sociodemographic data will also be compared between treatment groups (Group 1: ipsi-DLPFC, Group 2: contra-CEREB, Group 3: ipsi-DLPFC + contra-CEREB, Group 4: SHAM) to identify potential collapse factors.

In order to quantify changes in motor performance with the visuo-motor adaptation task,and assess tDCS effects applied over the cerebellum or the dorsolateral prefrontal areas on upper limb voluntary motion, the following set of measures will be used: (i) the angular trajectory error (“SumErr”; cm) estimating accuracy, consisting of the sum of observed deviations from the ideal linear trajectory at peak tangential velocity (PV)for each trial of the motor adaptation block; (ii) the area under the learning curve (AUC) measuring patients’ adaptation rate,calculated by performing a power fit on the mean learning values of each participant; (iii) the coefficient of variation (CV)measuring the variability of motor performance during the adaptation plateau phase (i.e., when the AUC is stabilized and reaches stable levels) and calculated as the standard deviation (SD) divided by the mean over trials belonging to the plateau phase (AUC/Time-to-target). All these metrics will be represented in ordinal and mean group scores for post vs. pre-tDCS baseline intervention, and for the follow-up milestone and compared across tDCS groups.

In order to quantify sustained attentional abilities (as a proxy of dorsolateral prefrontal  modulation by tDCS) with the AX-CPT task, we will analyse the total number of errors used as a proxy of sustained attention failure. An ordinal value will be taken and group means will be compared between pre- vs. post-tDCS  and for the follow-up milestone. In order to indentify and quantify changes of visuo-spatial performance (as a proxy of visuo-spatial orienting and selective attentional disability), the number of  crossed/circled letters (letter cancellation test) or bells (Bells cancellation test) and the shift (in mm and % of the total line length) of manual bisections from the precise midpoint of each line (line bisection test) will be analysed. Mean group total ordinal scores will be compared between groups acrossthe different time points (pre-, post-tDCS s and follow-up milestones). Other unanticipated complementary metrics could also be taken into account for the final analyses.

### EEG data analysis

EEG data will be preprocessed using a hierarchically organized pipeline proceeding as follows [59]. Data will be first segmented into contiguous epochs after a preliminary investigation on which epoch length is the most robust for analyses. Then, a 2nd-order infinite Butterworth forward and backward filter with a 0.5–45 Hz low-pass high-pass filter at a resolution of 2 Hz (500 ms time windows) with a Hanning taper window will be applied. Eye movements or muscle-related artifacts will be removed using a common Independent Component Analysis (ICA), supervised and manually corrected after expert visual verification. Epochs with signals exceeding 120μv peak-to-peak amplitude will be also removed. Finally, channels will be re-referenced to their common average. Once data pre-processing is completed, (i) power spectrum density (PSD), (ii) connectivity, and (iii) complexity measures will be calculated. These approaches will be individually conducted on each subject for every condition or task (resting-state recordings, visuo-motor adaptation task, AX-CPT task, associated to everytime point of our study timeline (baseline, post-tDCS, follow-up).

 A time-frequency analysis will be conducted in order to explore pre tDCS (baseline) vs. post  tDCS and vs. follow-up tDCS-related modulations of patients’ oscillatory activity. To this end, the amplitude of the power spectrum density ($\mu {V}^{2}$) for each frequency band (δ: 0.5–4 Hz; θ: 4–8 Hz; α: 8–12 Hz; β: 12–30 Hz; γ: 30–60 Hz) will be computed by means of a Fast Fourier Transformation (FFT) using Welch’s method with a 500-ms periodogram. Relative and absolute spectral normalized power (NP) will be then extracted for each EEG electrode. Normalized average power for all frequency bands will be computed and plotted to explore differences between pre-tDCS (baseline) vs. post-tDCS and vs. follow-up milestones within each group and to compare differencesacross independent groups stimulated with different tDCS approaches.

Functional connectivity analyses by means of EEG sensor level coherence measures will be conducted to explore the “reshaping” of intra-hemispheric and inter-hemispheric connectivity (assessing functional integration and segregation mechanisms at the network level) by tDCS approaches. Given that resting-state data can be influenced by false positive correlations and to avoid the impact of volume conduction on raw coherence measures, we will compute more specifically the imaginary part of coherence (ImCoh) [60] by means of a Fast Fourier Transformation (FFT). Intra-hemispheric functional connectivity analysis will focus on synchronization measures between ipsilesional DLPFC and M1/premotor (electrodes and F3-C3/F4-C4) and also ipsilesional DLPFC and parietal systems (electrodes F3-P3/F4-P4). For inter-hemispheric functional connectivity, our analyses will focus on synchronization measures between premotor/M1 (electrodes C3–C4) and also prefrontal systems (electrodes F3–F4). Overall, an 8 × 8 connectivity matrix contemplating all connections between all possible electrode combinations, will be explored.

Complexity analyses will explore tDCS-related changes of neuroplastic properties and estimate the predictability of EEG signals following a given tDCS condition by calculating multiscale sample entropy (MSE). This estimate quantifies the probability that neighboring data points may be in a predetermined range in a time series $$\{{x}_{1}, {x}_{2},...{x}_{N}\}$$, computing how often patterns re-occur in a time-domain sample (i.e., temporal irregularity prediction in the time-domain). The computation of MSE is divided into two steps: (1) first, a moving-averaging procedure is computed to express the dynamic representation of a system; (2) Second, the degree of predictability is measured for each of the moving-averaged time series $${z}^{r}$$ by means of the sample entropy ($${Sample}_{En})$$ method (see [61] for a complete formulation of MSE).

### MRI data analysis

MRI T1 sequence will be used to characterize stroke lesion features. To this end, a lesion overlay in normalized space for the complete sample of MCA stroke participants will be compiled. First, brain lesions will be manually drawn outlining damaged areas directly on the T1-weighted MRI sequences in native space using MRIcron software (v1.0.2.), outlining the precise anatomical boundaries of the stroke lesion. A graphic tablet (WACOM One) will be used for lesion mask delineation by an expert researcher (co-author XC-T) trained in neuroimaging and neuroanatomy upon advice from additional co-investigators (co-authors AV-C, MT, and MTC). Then, lesion masks will be normalized in SPM12 using a unified segmentation method depicting co-registered image lesions overlapped in a template atlas. Further, according to parcellated cortical structures based on Brodmann areas, the percentage of impacted voxels within each parcel and damaged tracts will be characterized and correlated with the set of above-mentioned outcome measures.

### Biophysical E-field models

As indicated above, electrode placement for the different tDCS conditions of our trial has been optimized by using a biophysical model of current distribution on a standard head/brain volume (software SimNIBS 3.2.3). Nonetheless, due to anatomical interindividual differences, the impact of electric currents at targeted structures (the DLPFC and the anterior lobe of the cerebellum) can slightly differ from subject to subject. For that reason, individual biophysical models will be generated to estimate the E-field (electrical current fields) strength distribution on each participant's MRI. On such basis, we will study *post hoc* the influence of model-generated variables (peak E-field strength at target, and the volume of anatomical layers the field needs to go through to reach the target) and their association with motor clinical recovery outcomes, the modulation of prefrontal (DLPFC) or cerebellar (CEREB) cognitive contributions and EEG outcome measures. To this end, individual T1-weighted MRI head models will be reconstructed for each patient. Tisue segmentation layers (air, bone, CSF, eyes, GM, WM, and skin) will be manually corrected with a graphic tablet by an experienced researcher (co-author XC-T) upon advice by expert co-investigator (co-authors AV-C and MT). After manual correction, head models will be re-reconstructed to ensure acceptable delineation of lesion and tissue boundaries. Then, tDCS current distribution and target peak E-field strength induced by the aforementioned electrode montages will be simulated using finite-element modeling (FEM) solving Laplace equation. To perform further voxel-level analysis correlations, individual E-field distribution will be normalized and its magnitude co-registered into a standard head model. Data extracted from individual models will be correlated to modeled peak currents on stimulated sites for each participant (ipsilesional DLPFC, contralesional CEREB, or the combination thereof) with the magnitude of motor recovery, cognitive modulations in dorsolateral prefrontal and cerebellar modulated cognitive outcomes, anatomical and lesion features. Additionally, we will correlate peak current density at cortical targets modelled individually with resting-state EEG measures.

## Statistical analyses

In order to evaluate the therapeutic potential of each tDCS intervention in post-stroke motor recovery, we will first asses the data distribution of the Fugl-Meyer Assessment by means of Shapiro–Wilk method. In the case of normal distribution, clinical outcomes will be compared using repeated measures analysis of variance (two-way ANOVA) with “TIME” (baseline, post-tDCS, follow-up) as within-subject factor, and “GROUP” (ipsi-DLPFC, contra-CEREB, ipsi-DLPFC + contra-CEREB, SHAM) as between-subject factor. Post hoc pair-wise comparisons will be performed using a Bonferroni correction for multiple comparisons. In the case of non-normal data distribution, a non-parametric Kruskal–Wallis test will replace repeated measures ANOVA. Linear mixed models and Pearson’s (for normally distributed data) or Spearman's correlation coefficients corrected for multiple comparisons between AX-CPT, visuo-motor adaptation task, and the Fugl-Meyer outcomes will be used to explore behavioral correlation between motor, attentional, and cognitive control domains. All data will be presented as mean ± standard deviation and statistical significance will be set for all tests at *p* ≤ 0.05.

In order to characterize the electrophysiological EEG effects induced by our interventions, we will first explore correlations with behavioral data and biomarkers of recovery. First, power spectral density (PSD) changes for each frequency band will be compared between stimulation groups using two-tailed ANOVA (*P* = 0.05), with “TIME” as the within-subject factor and “GROUP” as the between-subject factor. To correct for multiple comparisons, non-parametric cluster-based permutation statistics with Montecarlo sampling (1000 permutations) will be applied, allowing the examination of global effects across all electrodes while controlling for multiple comparisons at the sensor level, without the need of prior assumptions about effect location. Finally, connectivity (imaginary part of coherence measures) and entropy effects (complexity of temporal dynamics) of the electrophysiological response will be also explored. The same statistical procedure as for PSD exploration applying repeated measures ANOVA with non-parametric cluster-based permutation statistics and MonteCarlo sampling (1000 permutation) will be used, with “TIME” as within factor, and “GROUP” as between factor. Individually averaged connectivity and entropy values (total averages) and individual topographical maps at the electrode level will be examined to detect specific regions sensible to the tDCS intervention.

The individual differences across the study follow-up for the Fugl-Meyer Assessment evaluating upper/lower limb motor recovery,visuo-motor adaptation task outcomes assessing cerebellar contributions to motor learning and the AX-CPT task assessing sustained attention and cognitive control will be correlated (Pearson’s or Spearman correlation coefficients, depending on data normality) with the individuals’ electric fields voxel-by-voxel magnitude (an underneath cut-off of 0.25 V/m in total field strength will be positioned as a threshold in the power to induce neuronal effects). Moreover, the |E| and |nE| extracted values and the total injected current will be also correlated with the same outcome measures. Otherwise, E-field components will be correlated with specific EEG features reaching statistical significance in the power spectrum density ($\mu {V}^{2}$), connectivity, and complexity analyses. Finally, multivariate regression models will be computed to explore correlations between electrophysiological responses ($\mu {V}^{2}$, imaginary coherence and entropy measures), induced E-field, and behavioral outcomes.

All data will be analyzed according to the intention-to-treat principle. The data of all patients with complete datasets (at W0, W3, and W7 time points ) will be analyzed according to their randomized tDCS group. All available data will be included in the analysis. Missing data will be imputed by means of a constrained longitudinal data analysis (cLDA) which will estimate unconditionally lost data.

## Discussion

The current study protocol (*E-Brain*) aims at assessing the immediate and longer-term clinical potential of ten accumulative sessions of anodal high-density tDCS in patients with upper limb motor disability during the chronic phase  of a unilateral middle cerebral artery stroke. The novelty of our protocol is that instead of aiming to directly modulate damaged motor areas or their contralesional homologues, it focuses on assessing the isolated or combined stimulation of two regions such as the ipsilesional prefrontal cortex (DLPFC) and the contralateral anterior cerebellar lobe (CEREB), not directly involved (as premotor and primary motor systems do) in the execution of motor activity, but instead contributing indirectly to such via the modulation of associated cognitive processes.

A high number of prior conventional NIBS stimulation studies using repetitive TMS or tDCS in post-stroke motor dysfunctions have based their interventions on (i) the upregulation (with anodal tDCS, high-frequency rTMS or iTBS patterns) of ipsilesional M1/premotor systems; (ii) the downregulation (with cathodal tDCS, low-frequency rTMS, or cTBS patterns) of spared contralesional M1/premotor systems by virtue of the trans-callosal rivalrous mutually inhibitory  interactions, remapping, reorganizing, or normalizing abnormal excitability levels in lesional and perilesional areas [62]; or (iii) the upregulation of motor control/coordination systems such as the supplementary motor area (SMA) or the cerebellum. Unfortunately, initial enthusiasm for many of these approaches tested in small clinical trials has dwindled due to the lack of consistent effects when evaluated in larger populations of patients, and efficacy remains debated [63]. For this reason, it is paramount to explore and provide proof-of-concept for new treatments based on the manipulation of cortical sites, with the ability to drive improvements by acting on spared 'non-purely motor ' regions indirectly contributing to the recovery of voluntary motion via the modulation of associated cognitive processes and network-synchronization mechanisms.

In the cognitive domain, anodal tDCS over the ipsilesional DLPFC and/or cathodal stimulation of the contralesional DLPFC have shown efficacy in the modulation of prefrontal functions such as sustained attention and cognitive control [64–66]. Likewise, the modulation of the anterior lobe of the cerebellum has shown promise in stroke patients, given its active role in motor learning, and motor coordination, and its ability to contribute to motor “reorganization” when premotor or primary corticospinal systems are severely damaged [32–34]. Additionally, the neural signature of middle cerebral artery damage has revealed interactions between alterations of sustained attention deficits and impairments of motor function, emphasizing the importance of inter-areal communication in motor rehabilitation [20], hence the need for restorative stimulation methodologies able to integrate large-scale network-wide synchronization mechanisms across these structures. However, limitations in terms of spatial resolution posed by clinical EEG with few recording sensors-useful to explore the clinical evolution of neurophysiological markers in individual patients-has limited our comprehension of the underlying processes subtending motor recovery in stroke. Moreover, technical limitations of the first generation of tDCS devices to implement multi-site stimulation (i.e., targeting with acceptable spatial resolution via high-density montages different cortical nodes simultaneously) have contributed to keeping 'network' neuromodulation approaches poorly explored.

In such context, the protocol *E-Brain* aims at comparing in separate and independent patient groups three active anodal tDCS conditions (ipsi-DLPFC, contra-CEREB, ipsi-DLPFC + contra-CEREB) and a SHAM tDCS intervention. Our design will contribute to identifying the most beneficial strategy comparing single-site (monofocal) or combined (bifocal) dual-site approaches by means of high-density tDCS. The study will also ascertain changes in DLPFC and anterior cerebellar lobe modulation by means of tasks specifically assessing sustained attention/cognitive control and visuo-motor adaptation skills respectively, to verify that upper limb motor improvements are mediated by the modulation of such contributing networks. Moreover, different EEG synchrony measures recorded along the intervention will report on local and “large-scale” modulations of primary motor systems from distant regions, via changes in functional connectivity and local and long-range synchronization mechanisms [67] and reveal the longer-term neuroplastic properties associated with such effects.

Still a key challenge for clinical neuroscience [68, 69], this approach is currently inspiring a transition from conventional “single-site” neuromodulation focused on targeting directly impaired systems, towards multifocal stimulation set-ups with multiple electrical sources able to address more holistically, network dysfunction [70, 71]. Moreover, it is supported for example by recent work demonstrating higher modulatory power on cortical reactivity [66] and changes in corticospinal excitability [72, 73] by dual-site (bifocal) stimulation as compared to single-site (monofocal) tDCS approaches. In this context, our protocol will be among the first fully adopting the notion of post-stroke motor paralysis as a network impairment involving not only motor but also non-motor cognitive systems contributing indirectly to voluntary motion. Under such perspective, we will pursue the modulation of network dysfunction via a circuit-based therapeutic approach and promote local and global synergistic network-wide effects (“reshaping”) between non-purely primary motor regions (such as the prefrontal cortex and the anterior cerebellar lobe) and motor and premotor systems to facilitate motor post-stroke rehabilitation and effective recovery.

Our protocol is however not exempt from risk and might suffer from potential limitations, which we will try to minimize. First, recovery of lost motor function occurs slowly and gradually. Thus, the ten sessions of stimulation and a monthly follow-up planned for our patients might not be sufficient to drive or account for significant motor skill re-acquisition. Nonetheless, a more intense (i.e., a higher number of daily and cumulated sessions over time) and longer-lasting regime or follow-up period is currently outside of the scope of our protocol and limited by the scarcity of available time and resources. Two weeks of stimulation and a single monthly post-treatment follow-up seems a reasonable compromise for an experimental trial that once proven potentially successful, can be developed at a larger scale. Second, stroke patients even if sharing the same cardinal symptom, upper hand motor paralysis, can be differently affected by types of lesional mechanisms, lesion volume and lesion location, impacting the severity of the impairments, their ability to fully understand and perform motor or cognitive tasks hence compromise the reliability of behavioral and EEG evidence. Therefore, even if the *MinimPy* randomization algorithm counterbalancing groups by sex, age, and stroke type (ischemic/hemorrhagic) should minimize such risk, interindividual variability in lesion extent and clinical severity might be not equally distributed across the four experimental groups, precluding comparability. Third and least, the “live” on-site and at home rehabilitation activities associated to this protocol could slightly differ across patients, whereas uncontrollable factors such inner motivation or the intensity of unregulated outside activities with rehabilitative value could interfere and mask the real impact of tDCS interventions. For these reasons, on-site “live” rehabilitation programs during the treatment will be rightly monitored to make sure patients’ programs are comparable across groups and kept unchanged. Additionally, patients will be regularly questioned with regard to their daily life activities during participation in the study and all their comments will be documented.

In sum, the alarming pandemic-like rates reached by the consequences of acquired brain damage in developed societies [74] and the limitations shown by conventional monofocal TMS or tDCS approaches in post-stroke motor rehabilitation calls for the design and assessment of novel treatments in experimental double-blind, controlled trials. Our protocol will test an innovative tDCS-based strategy, easy to implement clinically, aiming at modulating associated structures contributing respectively to sustained attention and cognitive control by dorsolateral prefrontal networks and also to visuo-motor adaptation by anterior lobe cerebellar regions. More generally, beyond pure clinical applications in motor improvement after an MCA stroke, our study is designed to provide insight on the anatomical and physiological foundations of motor impairments and tDCS neuroplastic phenomena driving recovery. Finally, if our intervention demonstrates clinical efficacy, our protocol will pave the way for the development of individually customized tDCS strategies based on multi-site interventions at a larger scale and the design of more sophisticated and better-adapted neuromodulation technologies.

## Trial status

The first version of the study protocol was approved by the local ethics committee at the Institut d’Investigació Sanitària Pere Virgili (IISPV, Tarragona, Spain) on July 9th 2021. A pilot group of *n* = 8 patients, which will not be included in the final analyses served to pilot the initial version of the protocol, hence to improve its design and content, and to test the adequacy, feasibility, and reliability of some of the assessment tasks, leading to the current final version. Participant recruitment began on March 2022; and since then, *n* = 11 participants have successfully completed the protocol. Participant recruitment is planned to be completed by end of December 2025.

### Supplementary Information

**Additional file 1.** Standard protocol items: recommendation for interventional trials (SPIRIT) 2013 checklist: recommended items to address in a clinical trial protocol and related documents.

**Additional file 2: Supplementary Table.** World health organization trial registration data set.

## Data Availability

Not applicable. No data is available at this point because authors are still in the process of gathering such.

## Abbreviations

AX-CPT: AX continuous performance task
BA: Brodmann area
CAT12: Computational anatomy toolbox 12
CEREB: Cerebellum
CNS: Central nervous system
Coords: Coordinates
CSF: Cerebrospinal fluid
contra-CEREB: Contralesional cerebellar stimulation
CT: Computed tomography
DAN: Dorsal attention network
DTI: Diffusion tensor imaging
DLPFC: Dorsolateral prefrontal cortex
EEG: Electroencephalography
E-field: Electric field
FMA: Fugl-Meyer assessment
fMRI: Functional magnetic resonance image
GM: Gray Matter
HD-tDCS: High-density transcranial direct current stimulation
Hz: Hertz
ipsi-DLPFC: Ipsilesional dorsolateral prefrontal cortex
mA: Milliamps
MCA: Middle cerebral artery
MNI: Montreal Neurological Institute
MoCA: Montreal cognitive assessment
MRI: Magnetic resonance imaging
NIBS: Non-invasive brain stimulation
NIHSS: National institute of health stroke scale
BSIdir: Pair-wise derived brain symmetry index
PET: Positron emission tomography
PSD: Power spectrum density
ROIs: Regions of interest
SMN: Sensory-motor network
SPM12: Statistical parametric mapping
tDCS: Transcranial direct current stimulation
tES: Transcranial electric stimulation
V/m: Volts per meter
W: Week
WM: White Matter
|E|: Electric field strength
|nE|: Normal component of the electric field
