# Supplementary material for: High-density transcranial direct current stimulation to improve upper limb motor function following stroke: study protocol for a double-blind randomized clinical trial targeting prefrontal and/or cerebellar cognitive contributions to voluntary motion
Source: Trials. 2023 Dec 4;24:783. doi: 10.1186/s13063-023-07680-8 (PMC10694989; doi:10.1186/s13063-023-07680-8)
Supplement: Supplementary file 2 — Additional file 2: Supplementary Table. World health organization trial registration data set. [file 13063_2023_7680_MOESM2_ESM.pdf]

WHO data set (version 1.3.1)	
**1. Primary Registry and Trial Identifying Number**	*Clinical, Electrophysiological and E-field modelling evidence of high-density transcranial direct current stimulation in motor stroke (E-BRAIN). ClinicalTrials.gov Identifier: NCT05329818*
**2. Date of Registration in Primary Registry**	*April 15, 2022*
**3. Secondary Identifying Numbers**	*Registration number to the local ethics committee Institut d'investigació sanitària Pere Virgili (IISPV, Tarragona, Spain): 077/2021 (version: V.1_06/05/2021).*
**4. Source(s) of Monetary or Material Support**	*This study is supported by the Universitat Rovira i Virgili, Tarragona, Spain (2021PFR-URV-91;2022PFR-URV-29, and by the Rehabilitation service of the Hospital Universitari Joan XXIII (Tarragona, Spain).*
**5. Primary Sponsor**	*Universitat Rovira i Virgili, Tarragona, Spain*
**6. Secondary Sponsor(s)**	*Rehabilitation service of the Hospital Universitari Joan XXIII (Tarragona, Spain).*
**7. Contact for Public Queries**	*Email address: mariateresa.colominas@urv.cat*<br>*Telephone number: +34 977 25 7895*<br>*Postal address: 43007 Tarragona, Spain*
**8. Contact for Scientific Queries**	*MariaTeresa Colomina Fosch*<br>*mariateresa.colominas@urv.cat*<br>*Full time Professor of Psychobiology*<br>*Postal address: 43007 Tarragona, Spain*<br>*Universitat Rovira i Virgili*<br>*Research Group in Neurobehavior and Health (NEUROLAB)*<br>*Research Center for Behavioral Assessment (CRAMC)*<br>*Center of Environmental Food and Toxicological Technology (TecnATox) http://www.tecnatox.cat*<br>*ORCID: https://orcid.org/0000-0002-5619-4874*
**9. Public Title**	*Brain stimulation for post-stroke neurological deficits: a clinical trial.*
**10. Scientific Title**	*Final version: High-density transcranial direct current stimulation to improve upper limb motor function following stroke: study protocol for a double-blind randomized clinical trial targeting prefrontal and/or cerebellar cognitive contributions to voluntary motion. Acronym: E-brain.*
**11. Countries of Recruitment**	*Spain*
**12. Health Condition(s) or Problem(s) Studied**	*Post-stroke participants with hemiplegia and cognitive decline*
**13. Intervention(s)**	*Four interventional groups are considered in the present clinical trial : ipsilesional DLPFC tDCS, contralesional cerebellar tDCS or combined DLPFC + contralesional cerebellar tDCS, and a sham tDCS intervention). All subjects will receive 10 sessions*

	(once a day x 2 weeks) of brain sitmualtion of 20 minutes each. Please see the methods section of the manuscript for detailed information.
**14. Key Inclusion and Exclusion Criteria**	*Inclusion criteria : (1) to have received a diagnosis of supratentorial ischemic or hemorrhagic unilateral stroke supplied by the middle cerebral artery (i.e., encompassing frontal-temporal-parietal territories); (2) to be enrolled in a 'live' rehabilitation program in the rehabilitation and physical medicine department of our institution; (3) to be between 18 and 85 years old; (4) to have suffered a stroke 4 and 12 months prior to enrollment; (5) to have signed the informed consent form.*<br>*Exclusion criteria : (1) Unstable medical condition (e.g., affected by infections, with assisted ventilation, having suffered or actively suffering epilepsy or recurrent seizures, untreated psychiatric disorders or being an active treatment with sedative drugs); (2) Participants presenting contraindications to tDCS according to the most current international safety guidelines ; (3) Participants presenting cognitive impairments -such as severe aphasia or neuropsychiatric deficits- limiting their comprehension and their ability to follow instructions*
**15. Study Type**	*We here present an interventional double-blind (participants and operators are blinded to group allocation), sham-controlled, single-center, randomized pilot clinical trial involving participants having suffered a unilateral middle cerebral artery (MCA) stroke resulting in motor paralysis of the contralateral upper limb.*<br>*The main primary purpose pursued in this clinical trial is to identify the clinical potential of a multitarget cortical stimulation protocol by assessing the comparative effect of three tDCS interventions (cerebellar stimulation, dorsolateral prefrontal stimulation and the combination thereof) driving clinical improvements of upper-limb motor function in stroke patients.*
**16. Date of First Enrollment**	*05/05/2022*
**17. Sample Size**	*Estimated total 80 participants (20 per group). Up to date, 19 subjects have been enrolled.*
**18. Recruitment Status**	*Recruiting*
**19. Primary Outcome(s)**	*The primary outcome will evaluate changes in the Fulg-Meyer assessment (FMA) for the impaired upper limb in a baseline evaluation (before starting the treatment), 2 days post-treatment and 30 days post-treatment.*
**20. Key Secondary Outcomes**	*A set of secondary outcome measures taken prior to and following tDCS treatment will assess the patient's performance in a series of computer-based behavioral*

	tasks evaluating and visuomotor adaptation (cerebellar contribution) performance and also sustained attention and cognitive control (prefrontal contribution); changes in resting-state and task-evoked EEG recordings and a set of clinical scales evaluating global stroke severity, cognitive impairment and their recovery and correlations with predicted electric field distribution model features and stroke lesion hallmarks revealed by structural MRI neuroimaging. All the tests will be conducted three times: in a baseline evaluation (before starting the treatment), 2 days post-treatment and 30 days post-treatment. Please see the methods section of the manuscript for detailed information.
**21. Ethics Review**	*The first version of the study protocol was approved by the local ethics committee Institut d'Investigació Sanitària Pere Virgili (IISPV, Tarragona, Spain) on 9 July 2021 (https://www.iispv.cat/)*
**22. Completion date**	*Estimated completion date 01/12/2024.*
**23. Summary Results**	*Up to date, the current study is recruiting participants. Primary and secondary outcomes will be statistically compared between intervention groups and results will be published in international peer-reviewed journals once the sample size will be completed.*
**24. IPD sharing statement**	*No*
